# Cellular Entry of Binary and Pore-Forming Bacterial Toxins

**DOI:** 10.3390/toxins10010011

**Published:** 2017-12-26

**Authors:** Alexey S. Ladokhin

**Affiliations:** Department of Biochemistry and Molecular Biology, University of Kansas Medical Center, Kansas City, KS 66160, USA; aladokhin@kumc.edu; Tel.: +1-913-588-0489

This Special Issue of *Toxins*, entitled “Cellular Entry of Binary and Pore-Forming Bacterial Toxins,” gives a sense of the recent advances in characterizing the functional and structural aspects of this broad scientific problem that goes beyond the classical field of toxinology and microbiology and spills into the general areas of biochemistry, biophysics, and molecular and cell biology. The contributions to this Special Issue include several experimental articles, employing sophisticated techniques to gain important insights into the mechanism of cellular entry [1,2,3,4,5,6]; a thought-provoking perspective comment [7]; and two conceptual reviews, one on apicomplexan pore-forming toxins [8] and one on clostridial binary toxins [9]. What have we learned about the field from this collection? Despite the limited selection, some general features can be identified.

*Deciphering complex pathways requires integration of various approaches*. Cellular entry of bacterial toxins utilizes a complex mechanism [8,9] that involves multiple protein partners interacting with each other [3,9] and with a lipid bilayer [1,2,6]. Key players often undergo profound conformational changes, both in aqueous [5] and membranous environments [1,2]. Characterizing these functionally important conformational changes is a prerequisite for deciphering the mechanisms of cellular entry on a molecular level. One of the biggest challenges in establishing the structure–function relationships for bacterial toxins lies in their environment-dependent conformational lability. Consequently, even if a high-resolution structure of the soluble conformation is well-characterized, the mechanism might remain elusive, due to conformational rearrangements triggered by environment acidification and membrane insertion, common for the endosome-dependent pathways. These challenges could be met, for example, by careful examination of site-directed mutagenesis with a variety of functional assays (e.g., for diphtheria toxin [6]), complemented with molecular modeling (e.g., for perfringolysin O [1]). In another example, a sophisticated combination of cryo-electron microscopy, performed on elaborately prepared nanodisc samples, and computer simulations is used to resolve the structure of the pore of the anthrax toxin protective antigen in a lipid environment and in a complex with the toxin’s lethal factor [2].

*Structured vs. unstructured passageways through the membrane*. Bridging cellular membranes is a key step in the pathogenic action of both binary and pore-forming toxins. The former use their translocation domains, containing various structural motifs, to ensure efficient delivery of the toxic component into the host cell, while the latter act on the cellular membrane itself. In either case, the integrity of the membrane is compromised via targeted protein–lipid and protein–protein interactions triggered by specific signals, such as proteolytic cleavage and/or endosomal acidification. Several studies presented in this Special Issue either explicitly describe the formation of the water-filled protein structures that span the lipid-bilayer or implicitly evoke such structures, as a required part of the cellular entry mechanism. Specific structural examples that include both binary (e.g., anthrax [2]) and pore-forming toxins (e.g., perfringolysin O [1]) involve the insertion of the β-strands from multiple protein subunits to form a barrel-like structure that bridges the lipid bilayer in a permanent way. A similar concept has been evoked for other toxins as well, the translocation domains of which form α-helices in the lipid bilayer. Specifically, the translocation domain of diphtheria toxin was often assumed to use the so-called open-channel state (OCS), formed by three transmembrane helices, as a translocation pathway. The examination of both in vivo and in vitro activity of the several OCS-blocking mutants, presented in this issue [6], revealed that the OCS is formed after the translocation, which is likely to utilize an unstructured and possibly transient passageway. Certainly, more studies with other toxins are needed before any general conclusions can be reached on the possible differences between the actions of toxins that utilize α-helical vs. β-structure motifs in their membrane-interacting domains. More studies are also needed to fully characterize the structural and thermodynamic aspects of the conformational switching and membrane interactions involved in the cellular entry of bacterial protein toxins. Deciphering the physicochemical principles underlying these processes is also a prerequisite for the use of protein engineering to develop toxin-based molecular vehicles capable of targeted delivery of therapeutic agents to tumors and other diseased tissues.

## References

[1] Savinov S.N., Heuck A.P. (2017). Interaction of Cholesterol with Perfringolysin O: What Have We Learned from Functional Analysis?. Toxins.

[2] Machen A.J., Akkaladevi N., Trecazzi C., O’Neil P.T., Mukherjee S., Qi Y., Dillard R., Im W., Gogol E.P., White T.A. (2017). Asymmetric Cryo-EM Structure of Anthrax Toxin Protective Antigen Pore with Lethal Factor N-Terminal Domain. Toxins.

[3] Tausch F., Dietrich R., Schauer K., Janowski R., Niessing D., Martlbauer E., Jessberger N. (2017). Evidence for Complex Formation of the *Bacillus cereus* Haemolysin BL Components in Solution. Toxins.

[4] Puri M., La Pietra L., Mraheil M.A., Lucas R., Chakraborty T., Pillich H. (2017). Listeriolysin O Regulates the Expression of Optineurin, an Autophagy Adaptor That Inhibits the Growth of *Listeria monocytogenes*. Toxins.

[5] Palma L., Scott D.J., Harris G., Din S.U., Williams T.L., Roberts O.J., Young M.T., Caballero P., Berry C. (2017). The Vip3Ag4 Insecticidal Protoxin from *Bacillus thuringiensis* Adopts A Tetrameric Configuration That Is Maintained on Proteolysis. Toxins.

[6] Ladokhin A.S., Vargas-Uribe M., Rodnin M.V., Ghatak C., Sharma O. (2017). Cellular Entry of the Diphtheria Toxin Does Not Require the Formation of the Open-Channel State by Its Translocation Domain. Toxins.

[7] Knap P., Tebaldi T., Di Leva F., Biagioli M., Dalla Serra M., Viero G. (2017). The Unexpected Tuners: Are LncRNAs Regulating Host Translation during Infections?. Toxins.

[8] Guerra A.J., Carruthers V.B. (2017). Structural Features of Apicomplexan Pore-Forming Proteins and Their Roles in Parasite Cell Traversal and Egress. Toxins.

[9] Takehara M., Takagishi T., Seike S., Oda M., Sakaguchi Y., Hisatsune J., Ochi S., Kobayashi K., Nagahama M. (2017). Cellular Entry of *Clostridium perfringens* Iota-Toxin and *Clostridium botulinum* C2 Toxin. Toxins.

